# Nutritional Care and Rehabilitation for Frailty, Sarcopenia, and Malnutrition

**DOI:** 10.3390/nu15234908

**Published:** 2023-11-24

**Authors:** Momoko Tohyama, Yuka Shirai, Yoji Kokura, Ryo Momosaki

**Affiliations:** 1 Department of Rehabilitation Medicine, Mie University Graduate School of Medicine, Tsu 514-8507, Japan; 323m005@o.mie-u.ac.jp (M.T.); yunnkaa@gmail.com (Y.S.); 2 Department of Nutrition, Hamamatsu Medicine University Hospital, Hamamatsu 431-3192, Japan; 3 Department of Nutritional Management, Keiju Hatogaoka Integrated Facility for Medical and Long-Term Care, Hosu 927-0023, Japan; yojikokura@hotmail.com

Frailty, sarcopenia, and malnutrition are highly prevalent among the older population. A previous study reported that 17% of older adults living in a community were frail, 14% suffered from sarcopenia, and 13% were afflicted by malnutrition [1]. Among older hospitalized patients, the number of affected people was even higher (47% were reported to be frail, 37% had sarcopenia, and 66% were afflicted with malnutrition) [2]. With an aging global population, the prevalence of these conditions is expected to increase. There are many complex factors that lead to frailty, sarcopenia, and malnutrition, including aging, weight loss, multimorbidity, and functional disability [3,4]. Therefore, cases of frailty, sarcopenia, and malnutrition may coexist.

Frailty, sarcopenia, and malnutrition have been associated with poor prognoses. In a study on patients admitted to an intensive care unit, frail patients had a higher risk of mortality and were less likely to be discharged than non-frail patients. Further, among hospitalized patients, frailty is a predictor of falls [5]. Sarcopenia was associated with mortality, functional decline, falls, and fractures in a previous meta-analysis [6], and malnutrition is considered a factor affecting length of hospital stay, falls, hospitalization complications, activities of daily living, quality of life, mortality, and hospital readmission [7]. Thus, these three conditions can negatively affect patients’ clinical outcomes and require immediate attention. In fact, they are reversible conditions that can be improved with appropriate interventions. Nutritional care and rehabilitation have been found to be effective for patients with frailty, sarcopenia, and malnutrition. For instance, nutritional interventions have been shown to have significant effects on physical function and mobility among older adults with frailty or pre-frailty [8]. Among malnourished hospitalized patients, nutritional interventions increased daily calorie and protein intake and weight gain, thereby reducing length of hospital stay and hospital admissions [9]. Randomized controlled trials examining the ability of nutritional interventions to decrease the risk of sarcopenia revealed an association with increased hand grip strength [10]. Exercise interventions for older adults with sarcopenia significantly improved muscle strength, balance, and muscle mass [11]. Resistance training effectively improved muscle strength, physical function, and body composition among older adults with frailty, pre-frailty, sarcopenia, or pre-sarcopenia [12]. In addition to studies on the effects of nutritional care and rehabilitation alone, it has been reported that combined exercise and nutritional interventions for older hospitalized patients ameliorate frailty and improve physical function [13]. For patients with possible malnutrition and sarcopenia, combined exercise and nutritional therapy improve physical function better than exercise alone [14]. Further, the combination of high energy intake and intensive rehabilitation was associated with functional recovery among acute stroke patients with sarcopenia [15]. Strength training, aerobic exercise, and energy supplementation (with high amounts of protein and vitamin D) were effective in treating frailty [16]. Management through nutritional care and rehabilitation may be a fundamental approach to treating frailty, sarcopenia, and malnutrition.

Screening and assessment are essential for providing nutritional care and rehabilitation to patients with or at high risk of frailty, sarcopenia, or malnutrition. The screening tools for frailty include the FRAIL scale and the Edmonton frail scale [17,18]. In addition, the cardiovascular health study index [19] and frailty index [20] are also major methods for assessing frailty. However, there are no uniform criteria for the diagnosis of frailty. The European Working Group on Sarcopenia in Older People 2 (EWGSOP2) recommended using SARC-F, a five-item questionnaire, as a screening tool for sarcopenia [21] and stressed the need to determine sarcopenia after screening by assessing muscle weakness, muscle quantity and quality, and the severity of sarcopenia via measuring physical function. There are several methods for measuring muscle strength, muscle mass, and physical function. The cutoff values for each measure may not be appropriate for the target region because of variability in patient characteristics such as height. Therefore, it is necessary to validate the cutoff value considering regional specificity and other factors. Moreover, the optimal index for assessing muscle quality remains unclear. Screening tools for malnutrition include the mini nutritional assessment-short form, malnutrition universal screening tools, and nutritional risk screening 2002 [7]. Although various nutritional screening tools have been validated, no reliable tools for all metabolic conditions have been identified. In addition, screenings must be simple, less invasive, valid, and reliable. It is necessary to verify whether screening tools are efficient in extracting patients while avoiding the requirement for excessive time and cost.

Despite the existence of various tools for screening and assessing frailty, sarcopenia, and malnutrition, uniform criteria and more convenient and reliable screening and assessment tools are needed. Uniform assessment methods would allow for the comparison of the prevalence, interventions, and outcomes of frailty, sarcopenia, and malnutrition worldwide. Further research is needed to validate the reliability of the screening and assessment methods employed. Nutritional care and rehabilitation have been reported to be effective for treating frailty, sarcopenia, and malnutrition. However, there is insufficient evidence on optimal intervention methods, improvements in clinical outcomes, cost-effectiveness, and the sustainability of improvements. Moreover, not all the causes of sarcopenia, frailty, and malnutrition are known. Thus, the underlying factors need to be investigated. Additional research is needed to determine the optimal interventions and their effects on preventing and ameliorating frailty, sarcopenia, and malnutrition.
